# 1*H*-2,3-Dihydroperimidine Derivatives: A New Class of Potent Protein Tyrosine Phosphatase 1B Inhibitors

**DOI:** 10.3390/molecules19010102

**Published:** 2013-12-23

**Authors:** Wen-Long Wang, Dong-Lin Yang, Li-Xin Gao, Chun-Lan Tang, Wei-Ping Ma, Hui-Hua Ye, Si-Qi Zhang, Ya-Nan Zhao, Hao-Jie Xu, Zhao Hu, Xia Chen, Wen-Hua Fan, Hai-Jun Chen, Jing-Ya Li, Fa-Jun Nan, Jia Li, Bainian Feng

**Affiliations:** 1 School of Pharmaceutical Science, Jiangnan University, Wuxi 214122, China; E-Mails: yangdlin@gmail.com (D.-L.Y.); yehuihua92@hotmail.com (H.-H.Y.); zhangsiqii@gmail.com (S.-Q.Z.); zyn0825@gmail.com(Y.-N.Z.); xhj223@hotmail.com (H.-J.X.); starryinsight@gmail.com (Z.H.); chenxia19891219@126.com (X.C.); 2 State key Laboratory of Drug Research, Shanghai Institute of Materia Medica, Chinese Academy of Sciences, Shanghai 201203, China; E-Mails: lxgao@mail.shcnc.ac.cn (L.-X.G.); cltang@mail.shcnc.ac.cn (C.-L.T.); wpma@mail.shcnc.ac.cn (W.-P.M.); chenhaij@gmail.com (H.-J.C.); jyli@simm.ac.cn (J.-Y.L.); fjnan@simm.ac.cn (F.-J.N.); 3 Jiangshu Alpha Biopharmaceuticals, Inc. Wuxi 214122, China; E-Mail: fanwenhua1987@163.com

**Keywords:** 1*H*-2,3-dihydroperimidine derivatives, PTP1B, inhibitors, selectivity, structure-activity relationships (SAR)

## Abstract

A series of 1*H*-2,3-dihydroperimidine derivatives was designed, synthesized, and evaluated as a new class of inhibitors of protein tyrosine phosphatase 1B (PTP1B) with IC_50_ values in the micromolar range. Compounds **46** and **49** showed submicromolar inhibitory activity against PTP1B, and good selectivity (3.48-fold and 2.10-fold respectively) over T-cell protein tyrosine phosphatases (TCPTP). These results have provided novel lead compounds for the design of inhibitors of PTP1B as well as other PTPs.

## 1. Introduction

Reversible protein tyrosine phosphorylation is a key regulatory mechanism in eukaryotic cell physiology [1]. Dysregulation of protein tyrosine kinases (PTKs) and protein tyrosine phosphatases (PTPs) is linked to numerous human diseases, including cancer, diabetes, obesity, infection, autoimmune, and neuropsychiatric disorders [2,3]. Hence, PTKs and PTPs are emerging as high value targets for therapeutic intervention [3,4,5,6,7]. Consequently, many efforts have been made to target these enzymes with small molecules in order to develop new therapeutic agents. Notable success has been achieved in targeting signaling pathways regulated by protein tyrosine phosphorylation with more than a dozen of small molecule kinase inhibitors on the market [8]. However, the therapeutic benefits of modulating PTPs are still underexplored despite the fact that several PTPs have been identified as high value targets [9].

Protein tyrosine phosphatase 1B (PTP1B), an intracellular nonreceptor PTPase, has received much attention due to its pivotal role in type II diabetes and obesity as a negative regulator of the insulin signaling pathway by dephosphorylating the activated insulin receptor [10]. Studies from two different laboratories have shown PTP1B-knockout mice exhibit enhanced insulin sensitivity, improved glucose tolerance and resistance to diet-induced obesity [11,12]. In addition, several groups have demonstrated that overexpression of PTP1B is sufficient to drive tumorigenesis in mice, providing additional support for the use of PTP1B inhibitors for cancer therapy [13,14]. Therefore, PTP1B seems to be a potential target for the treatment of type 2 diabetes mellitus, obesity, and cancer. A variety of PTP1B inhibitors have been disclosed among academic and industrial laboratories [15,16,17]. Two compounds, ertiprotafib and trodusquemine, have progressed to clinical trials [18,19]. However, ertiprotafib was discontinued in phase II clinical trials due to a lack of efficacy and side effects [20]. There are two significant challenges to develop orally bioavailable, small molecular PTP1B inhibitors [15]: (1) it is difficult to design inhibitors that are specific for PTP1B due to the close homology with other PTPs, for example, T-cell protein tyrosine phosphatase (TCPTP) shares a structurally very similar active site with PTP1B and is about 80% homologous in the catalytic domain [21], and (2) many small molecules that bind with high affinity in active site are hydrophilic, and as a result they have poor cell permeability. Therefore, imminent development of potent and PTP1B specific inhibitor remains necessary.

**Figure 1:**
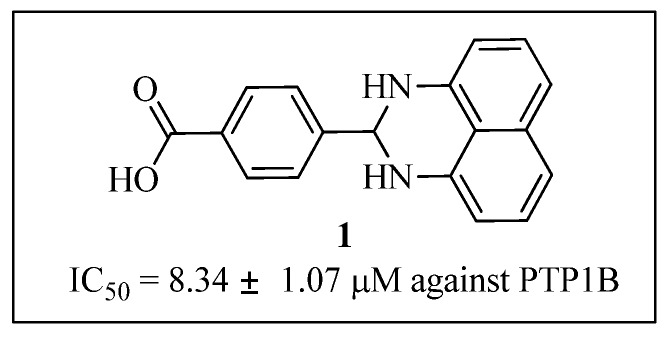
Chemical structure of hit compound **1**.

In searching for novel PTP1B inhibitors, we identified 4-(2,3-dihydro-1*H*-perimidin-2-yl)benzoic acid (**1**) as a novel PTP1B inhibitor (IC_50_ = 8.34 ± 1.07 μM) through high throughput screening of our compound collection (Figure 1). This result provided us a chance to explore novel small molecule inhibitors of PTP1B. Herein, we designed, synthesized a series of 1*H*-2,3-dihydroperimidine derivatives, evaluated their inhibitory activities toward PTP1B , and elucidated the SARs. Selected compounds were also subjected to selectivity analyses to determine whether their biological properties made them suitable for further development.

## 2. Results and Discussion

### 2.1. Chemistry

#### 2.1.1. Design of 1*H*-2,3-Dihydroperimidine Derivatives

Based on the structure of compound **1**, **42** new 1*H*-2,3-dihydroperimidine derivatives (compounds **2**–**6**, **8**–**19**, **21**–**35**, and **40**–**49**, Table 1) were designed and synthesized. Replacing the carboxylic acid moiety in compound **1** with bromo-, hydroxyl-, fluoro-, cyano-, or amino groups, we obtained analogues **2**–**6** and **8**. By coupling the carboxylic acid on compound **1** with a series of amino acids esters, compounds **10**–**14** were obtained. After saponification, we got the corresponding acid compounds **15**–**19**. By replacing the amide bond in compounds **15**–**19** with an oxygen atom we got ether derivatives **20**–**25**. Using amine derivative **8** as starting material, amide compounds **26**–**35** were obtained. We also synthesized compounds **40**–**49** by replacement of phenyl ring on compound **1** with pyridinyl ring.

#### 2.1.2. Synthesis of 1*H*-2,3-Dihydroperimidine Derivatives

**Scheme 1:**
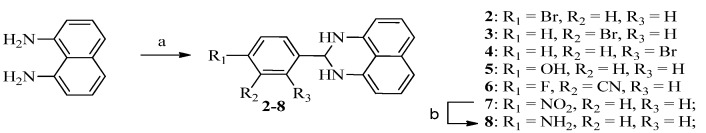
Synthesis of Compounds **2**–**8**.

**Scheme 2:**
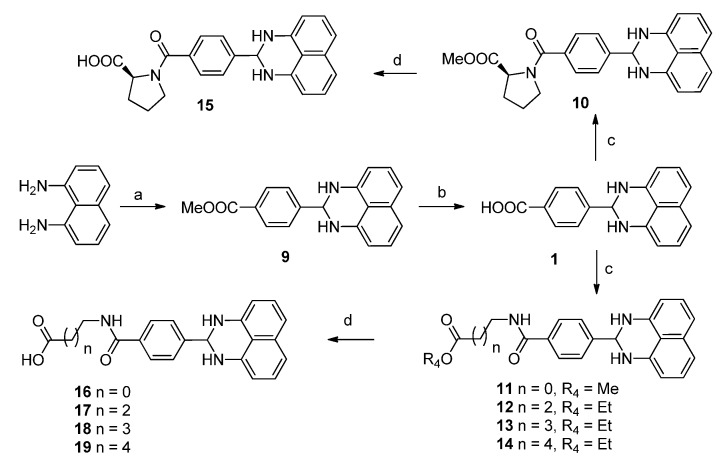
Synthesis of Compounds **10**–**19**.

**Scheme 3:**
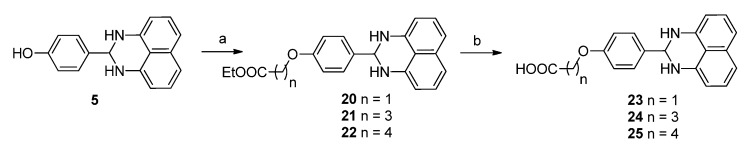
Synthesis of Compounds **20**–**25**.

**Scheme 4:**
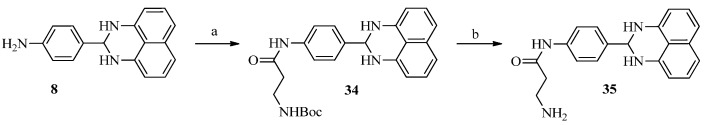
Synthesis of Compounds **26**-**33**.

**Scheme 5:**
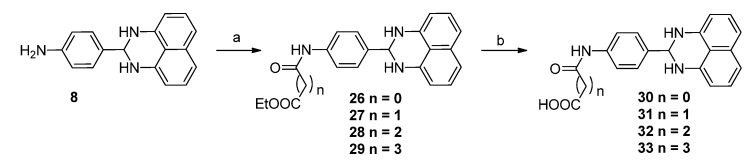
Synthesis of Compounds **34**–**35**.

**Scheme 6:**
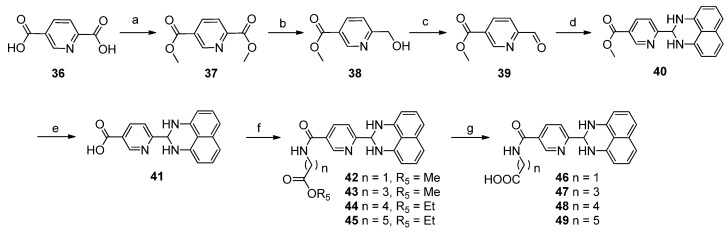
Synthesis of Compounds **40**–**49**.

The coupling reactions of 1,8-diaminonaphthalene with various aldehydes were accomplished in the presence of a catalytic amount of Zn(OAc)_2_ to yield compounds **2**–**7** (Scheme 1) and **9** (Scheme 2) in 31%–55% yields [22]. After reduction of nitro compound **7**, amine **8** was obtained in 89% yield. Compound **1** was obtained from compound **9** in 53% by saponification with lithium hydroxide in aqueous THF, followed by coupling with appropriate amino acid esters to yield compounds **10**–**14** in 35%–52% yield. After saponification with lithium hydroxide in aqueous THF, compounds **15**–**19** were obtained in 42%–55% yield (Scheme 2). Compounds **20**–**22** were prepared by alkylation of the phenol **5** with a series of bromo ethyl esters, followed by saponification with lithium hydroxide in aqueous THF to yield **23**–**25** in 35%–54% yield (Scheme 3). Amide compounds **26**–**29** were obtained in 35%–51% yield by acylation with ethyl chlorooxoacetate or various ethyl ester acids, followed by saponification with lithium hydroxide in aqueous THF to yield **30**–**33** in 35%–56% yield (Scheme 4). Compound **34** was prepared by coupling amine **8** with 3-((*tert*-butoxycarbonyl)amino)propanoic acid in the presence of EDCI and DMAP, followed by deprotection of the Boc group using TFA in CH_2_Cl_2_ to yield the amino compound **35** (Scheme 5). Scheme 6 describes the straightforward synthesis of the derivatives **40**–**49**. Obtained from compound **36** in the presence of SOCl_2_ using MeOH as solvent, diester **37** was selectively reduced to compound **38** in the presence of NaBH_4_/CaCl_2_[23]. Compound **38** was transformed into aldehyde **39** using Dess-Martin periodinane in the presence of CH_2_Cl_2_, followed by coupling with naphthalene-1,8-diamine to yield compound **40**, using similar conditions to those in Scheme 1. Then compounds **41**–**49** were obtained using similar conditions to those shown in Scheme 2.

*Reagents and conditions*: a—MeOH, Zn(OAc)_2_, aldehyde, overnight, 45%–55%; b—Fe, NH_4_Cl, EtOH/H_2_O, 89%.

*Reagents and conditions*: a—MeOH, Zn(OAc)_2_, 4-formylbenzoic acid methyl ester, overnight, 31%; b—LiOH, THF/H_2_O, 3 h, 53%; c—amino acid esters, EDCI, DMAP, 40 °C, overnight, 35%–52%; d—LiOH, THF/H_2_O, 42%–55%.

*Reagents and Conditions*: a—Br(CH_2_)_n_COOEt, Cs_2_CO_3_, 40 °C overnight, 40%–65%; b—LiOH, THF/H_2_O, 35%–54%.

*Reagents and Conditions*: a—ClCOCOOEt, Et_3_N, DCM, 41% or EtOCO(CH_2_)_n_COOH, EDC, DMAP, DMF, 35%–51%; b—LiOH, THF/H_2_O, 35%–56%.

*Reagents and Conditions*: a—BocNH(CH_2_)_2_COOH, EDCI, DMAP, DMF, 43%; b—TFA, DCM, 67%.

*Reagents and Conditions*: a—MeOH, SOCl_2_, 70 °C, 4 h, 98%; b—CaCl_2_, NaBH_4_, THF/EtOH, 0 °C, 58%; c—Dess-Martin periodinane, DCM, overnight, 86%; d—naphthalene-1,8-diamine, MeOH, Zn(OAc)_2_, overnight, 27%; e—LiOH, THF/H_2_O, 43%; f—NH_2_(CH_2_)_n_COOR_5_, EDCI, DMAP, DMF, 40%–60%; g—LiOH, THF/H_2_O, 42%–50%.

### 2.2. Biological Activities

#### 2.2.1. PTP1B Inhibitory Activities and Structure-Activity Relationships

The inhibitory activities of all the synthesized compounds against PTP1B were measured using *p*-nitrophenyl phosphate (pNPP) as substrate [24,25], and the results are detailed in Table 1. We initially prepared **2**–**6** and **8** by the route outlined in Scheme 1, in which we substituted the carboxylic acid group on the phenyl ring with bromo-, fluoro-, hydroxyl-, amino-, or cyano groups. We noticed that none of them showed more than 50% of enzyme inhibition against PTP1B at the concentration of 20 μg/mL. This result indicated the importance of the carboxylic acid for activity. As for compounds **9**–**14**, ester compounds showed poor enzyme inhibitory activity at the concentration of 20 μg/mL. Saponification of ester compounds **9**–**14** to the corresponding acid compounds **1** and **15**–**19** dramatically improved PTP1B inhibitory activity. These results further confirmed that the acid group was important for PTP1B inhibitory activity. Among compound **1** and **16**–**19**, compound **16** with an acetic acid moiety and compound **17** with a butanoic acid moiety showed three times less potency than compound **1**. Compound **18** with a pentanoic acid moiety and compound **19 **with a hexanoic acid moiety showed similar activity to compound **1**. Compound **15** with a proline moie ty exhibited similar activity to compound **16**. The results indicated that the distance between phenyl ring and carboxylic acid had some impact on the inhibitory activity. As for ether compounds **21**–**25**, ester compounds **21** and **22** did not show inhibitory activity.

Compound **23** with an acetic acid moiety exhibited slightly better inhibitory activity than compound **1**. However, compound **24** with butanoic acid moiety and compound **25** with pentanoic acid moiety showed no inhibitory activity. These results indicated that using O atom as linker between phenyl ring and carboxylic acid generally decreased the enzyme inhibitory activity and that the distance between carboxylic acid and phenyl ring impacted the enzyme inhibitory activity. As for amide compounds **26**–**33**, ester compounds **27**–**29** did not show activity, however it was interesting that compound **26** with an ethyl 2-oxoacetate moiety showed six times more potent enzyme inhibitory activity than compound **1**. Among acid compounds **30**–**33**, compound **30** with a 2-oxoacetic acid moiety and compound **32** with a 4-oxobutanoic acid had no activity against PTP1B. Compound **31** with a 3-oxopropanoic acid and compound **33** with a 5-oxopentanoic acid showed similar activity to compound **1**. Compound **34** and **35** showed no inhibitory activity against PTP1B.

**Table 1:** PTP1B inhibitory activities of compounds **1**–**6**, **8**–**19**, **21**–**35** and **40**–**49**. 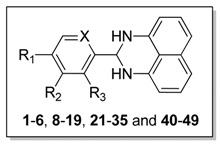

Comp	R_1_	R_2_	R_3_	X	Inhibition(%) at 20 μg/mL	IC_50_(μM) ^a^
**1**	COOH	H	H	CH	98.63%	8.34 ± 1.07
**2**	Br	H	H	CH	37.00%	NT ^b^
**3**	H	Br	H	CH	42.51%	NT
**4**	H	H	Br	CH	17.52%	NT
**5**	OH	H	H	CH	7.18%	NT
**6**	F	CN	H	CH	31.58%	NT
**8**	NH_2_	H	H	CH	23.76%	NT
**9**	COOMe	H	H	CH	15.27%	NT
**10**	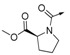	H	H	CH	26.25%	NT
**11**	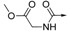	H	H	CH	4.81%	NT
**12**	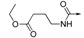	H	H	CH	6.83%	NT
**13**	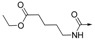	H	H	CH	28.48%	NT
**14**	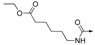	H	H	CH	35.57%	NT
**15**	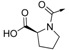	H	H	CH	84.21%	20.23 ± 1.94
**16**	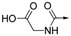	H	H	CH	89.99%	27.75 ± 5.45
**17**	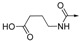	H	H	CH	73.13%	22.21 ± 1.60
**18**	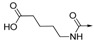	H	H	CH	94.84%	5.53 ± 0.54
**19**	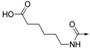	H	H	CH	95.35%	7.82 ± 0.27
**21**	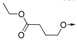	H	H	CH	7.45%	NT
**22**	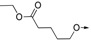	H	H	CH	2.04%	NT
**23**	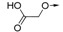	H	H	CH	98.39%	5.88 ± 0.25
**24**	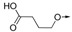	H	H	CH	10.48%	NT
**25**	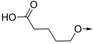	H	H	CH	32.13%	NT
**26**	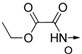	H	H	CH	91.76%	1.27 ± 0.06
**27**	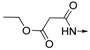	H	H	CH	1.68%	NT
**28**	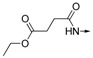	H	H	CH	29.69%	NT
**29**	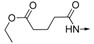	H	H	CH	2.92%	NT
**30**	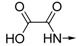	H	H	CH	0.51%	NT
**31**	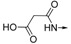	H	H	CH	96.83%	6.45 ± 0.42
**32**	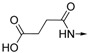	H	H	CH	29.95%	NT
**33**	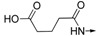	H	H	CH	95.07%	6.91 ± 1.17
**34**	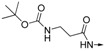	H	H	CH	12.61%	NT
**35**	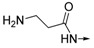	H	H	CH	0.29%	NT
**40**	COOMe	H	H	N	6.40%	NT
**41**	COOH	H	H	N	96.62%	10.82 ± 0.69
**42**	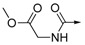	H	H	N	42.12%	NT
**43**	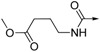	H	H	N	4.67%	NT
**44**	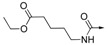	H	H	N	4.74%	NT
**45**	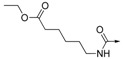	H	H	N	10.75%	NT
**46**	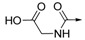	H	H	N	99.47%	0.66 ± 0.03
**47**	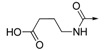	H	H	N	98.47%	15.24 ± 1.41
**48**	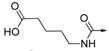	H	H	N	91.47%	3.56 ± 0.13
**49**	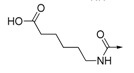	H	H	N	99.40%	0.59 ± 0.05
**OA ^c^**	-	-	-	-	-	2.41 ± 0.35

^a^ The pNPP assay. IC_50_ values were determined by regression analyses and expressed as means ± SD of three replications; ^b^ NT means not tested; ^c^ OA means oleanolic acid as positive control.

By replacing phenyl ring on compound **1** with a pyridinyl ring, ester compounds **40** and **42**–**45** did not show inhibitory activity. As for acid compounds, compound **41** showed similar activity to hit compound **1**. Compound **46** with acetic acid moiety and compound **49** with a hexanoic acid moiety showed submicromolar inhibitory activity, about fourteen times more potent than compound **1**. Compound **48** with a pentanoic acid moiety had inhibitory activity with IC_50_ of 3.56 ± 0.13 μM. Compound **47** with a butanoic acid moiety showed poorest inhibitory activity with IC_50_ of 15.24 ± 1.41 μM, about twenty-five times less potent than compounds **46** and **49**. The results indicated that the distance between pyridinyl ring and carboxylic acid was important for enzyme inhibitory activity, and that the replacement of phenyl ring with a pyridinyl ring obviously impacted on the enzyme inhibition.

#### 2.2.2. Selectivity against Other PTPs

In addition to potency improvements, we investigated the selectivity of three representative compounds, namely, **1**, **46** and **49** against other PTPs (TCPTP, SHP-1, SHP-2, LAR). As shown in Table 2, homogeneous T-cell protein tyrosine phosphatase (TCPTP) inhibitory activities were investigated simultaneously by the same method [24,25]. Compounds **46** and **49** showed 3.48-fold and 2.10-fold greater selectivity for PTP1B than for TCPTP respectively, while hit compound **1** exhibited poor selectivity with 0.58-fold for PTP1B than for TCPTP. Besides TCPTP, we tested the inhibitory activity of these compounds on other three homogenous enzymes SHP-1, SHP-2, and LAR [25]. As shown in Table 2, we concluded that compounds **46** and **49** had no visible activities against LAR, and compound **46** possessed about 9-fold selectivity for PTP1B over SHP-1 and SHP-2, while **49** showed about 4-fold selectivity for PTP1B over SHP-1 and SHP-2.

**Table 2:** The IC_50_ values of compounds **1**, **46** and **49** against PTPs.

Comp	IC_50_(μM) ^a^		TCPTP/PTP1B	IC_50_(μM) ^a^		
	PTP1B	TCPTP		SHP-1	SHP-2	LAR
**1**	8.34 ± 1.07	4.83 ± 0.90	0.58	23.31 ± 2.03	31.21 ± 7.72	NA^b^
**46**	0.66 ± 0.03	2.30 ± 0.17	3.48	6.01 ± 0.20	5.95 ± 0.29	NA
**49**	0.59 ± 0.05	1.24 ± 0.12	2.10	2.72 ± 0.30	2.50 ± 0.22	NA
**PC ^c^**	2.41 ± 0.35	5.14 ± 0.77		58.34 ± 1.96	36.65 ± 4.46	58.34 ± 1.96

SHP-1, SH2-Containing Protein Tyrosine Phosphatase-1; SHP-2, SH2-Containing Protein Tyrosine Phosphatase-2; LAR, leukocyte antigen-related tyrosine phosphatase; ^a^ The *p*NPP substrate and 3-o-methylfluorescein phosphate (OMFP) substrate were utilized in PTP1B/TCPTP assay, and SHP-1/SHP-2/LAR assay, respectively; IC_50_ values were determined by regression analyses and expressed as means ± SD of three replications; ^b^ NA: No activity (compound inhibitory ratio lower than 50% at the dose of 20 μg/mL); ^c^ PC: positive control; Oleanolic acid was for PTP1B and TCPTP, Na_3_VO_4_was for SHP-1, SHP-2 and LAR.

#### 2.2.3. Characterization of the Inhibitor on Enzyme Kinetics and Cellular Activity

**Figure 2:**
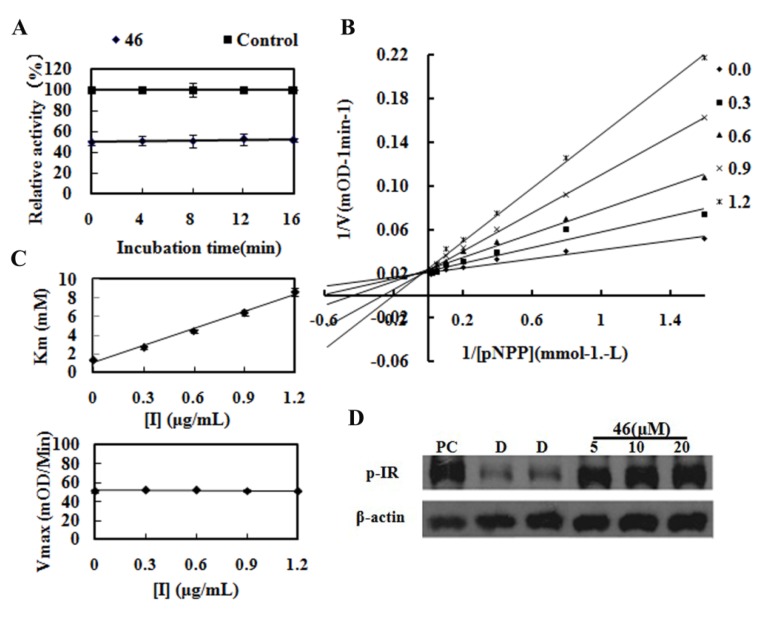
Characterization of **46** to PTP1B. (**A**) Fast-binding inhibition of PTP1B by **46**;(**B**) Typical competitive inhibition of **46** shown by Lineweaver-Burk plot; (**C**) The initial velocity determined with various concentrations of pNPP at various fixed concentrations of **46**; and (**D**) Effect of **46** on tyrosine phosphorylation of insulin receptor β in CHO/*h*IR cells.

A kinetic study was performed in order to identify the inhibitory mechanism of compound **46** (Figure 2), using the reported enzyme kinetics assays [25].

As shown in Figure 2A, **46** demonstrated a fast-binding inhibition of PTP1B. The fast-binding inhibition of **46** toward PTP1B may also exclude that **46** is a nonspecific inhibitor, because nonspecific inhibitors always show time-dependent behavior and steep inhibition curve [26]. We further determined the inhibition modality of **46** which inhibited PTP1B with the characteristics typical of a competitive inhibitor, as indicated by increased *k_m_* values and unchanged *V_max_* values when the inhibitor concentration was increased (Figure 2C). Meanwhile, the result of the Lineweaver-Burk plot confirmed **46** as a competitive inhibitor of PTP1B for intersecting at *y*-axis of a nest of lines with increased inhibitor concentration (Figure 2B). The results indicated that **46** binds the catalytic pocket of PTP1B and behaves as a competitor to the substrate.

To explore the effect of PTP1B inhibitor *in vitro* on insulin signaling, CHO cells overexpressing human insulin receptor (CHO/*h*IR) were treated in presence or absence of compound, then tyrosine phosphorylation level of insulin receptor (p-IR) was detected after stimulated by insulin. Compared with negative control of DMSO, compound **46** ranging from 5 µM to 20 µM greatly improved p-IR level. This result indicates that compound **46** shows well membrane permeability and could protect insulin pathway signaling on the cellular level (Figure 2D).

## 3. Experimental

### 3.1. Chemistry

All chemicals were reagent grade and used as purchased. All reactions were performed under an inert atmosphere of dry argon or nitrogen using distilled dry solvent. ^1^H (400 MHz) NMR spectra were recorded on a Bruker AVⅢ 400 MHz spectrometer. The chemical shifts were reported in (ppm) using the 7.26 signal of CDCl_3_ (^1^H-NMR) and the 2.50 signal of DMSO-*d*_6_ (^1^H-NMR) as internal standards and the 39.50 signal of DMSO-*d*_6_ (^13^C-NMR) as internal standards. ESI Mass spectra (MS) were obtained on a Waters Micromass Platform LCZ Mass Spectrometer. Melting points were recorded on YRT-3 melting point apparatus (Tianjin Reliant Instrument Co., Ltd., Tianjin, China) and were reported without correction.

#### 3.1.1. Procedure for the Preparation of Compounds **2**–**7**

To a stirred solution of 4-bromobenzaldehyde (421.8 mg, 2.28 mmol) in methanol (5 mL) was added a solution of naphthalene-1,8-diamine (300 mg, 1.90 mmol) in methanol (5 mL), followed by Zn(OAc)_2_·H_2_O (3.5 mg, 0.016 mmol). Then the mixture was stirred at room temperature for 16 h. The reaction mixture was filtered. The filter cake was washed with methanol, dried to get compound **2** (277 mg, 45%) as a brown solid, mp 138.9–142.3 °C. ^1^H-NMR (CDCl_3_) *δ*: 4.68 (brs, 2H), 5.45 (s, 1H), 6.54 (dd, *J* = 1.6 Hz, 6.4 Hz, 2H), 7.25(m, 4H), 7.52 (d, *J* = 8.4 Hz, 2H), 7.58 (d, *J* = 8.4 Hz, 2H); MS (ESI): *m/z* calcd. for C_17_H_14_BrN_2_[M+H]^+^ 325.0/327.0, found: 325.2/327.5.

*2-(3-Bromophenyl)-2,3-dihydro-1H-perimidine* (**3**). Yield = 48%, mp 161.7–163.3 °C; ^1^H-NMR (CDCl_3_) δ: 4.51 (brs, 2H), 5.44 (s, 1H), 6.54 (dd, *J* = 1.6 Hz, 6.8 Hz, 2H), 7.23–7.34 (m, 5H), 7.55–7.59 (m, 2H), 7.83 (s, 1H); MS (ESI): *m/z* calcd. for C_17_H_14_BrN_2_ [M+H]^+^ 325.0/327.0, found: 325.5/327.5.

*2-(2-Bromophenyl)-2,3-dihydro-1H-perimidine* (**4**). Yield = 46%, mp 130.4–131.0 °C;^1^H-NMR (CDCl_3_) δ: 4.62 (s, 2H), 5.94 (s, 1H), 6.58 (d, *J* = 7.2 Hz, 2H), 7.23–7.30 (m, 5H), 7.39 (t, *J* = 7.6 Hz, 1H), 7.61 (d, *J* = 8.0 Hz, 1H), 7.84 (dd, *J* = 1.2 Hz, 7.6 Hz, 1H); MS (ESI): *m/z* calcd. for C_17_H_14_BrN_2_ [M+H]^+^ 325.0/327.0, found: 325.4/327.7.

*4-(2,3-Dihydro-1H-perimidin-2-yl)phenol* (**5**). Yield = 52%, mp 148.1–152.2 °C; ^1^H-NMR (DMSO-*d_6_*) δ: 5.25 (s, 1H), 6.48 (d, *J* = 7.2 Hz, 2H), 6.62 (s, 2H), 6.82 (d, *J* = 8.0 Hz, 2H), 6.98 (d, *J* = 8.4 Hz, 2H), 7.14 (dd, *J* = 7.6 Hz, 8.0 Hz, 2H), 7.41(d, *J* = 8.4 Hz, 2H), 9.54 (s, 1H); MS (ESI): *m/z* calcd. for C_17_H_15_N_2_O[M+H]^+^ 263.1, found: 263.2.

*5-(2,3-Dihydro-1H-perimidin-2-yl)-2-fluorobenzonitrile* (**6**). Yield = 55%, mp 204.4–208.4 °C; ^1^H-NMR (DMSO-*d_6_*) δ: 5.44 (s, 1H), 6.50 (d, *J* = 7.6 Hz, 2H), 6.90 (s, 2H), 7.01(d, *J* = 7.6 Hz, 2H), 7.17 (dd, *J* = 7.6 Hz, 8.0 Hz, 2H), 7.60 (t, *J* = 9.2 Hz, 1H), 7.99 (dt, *J* = 2.4 Hz, 6.8 Hz, 1H), 8.14 (dd, *J* = 2.4 Hz, 6.4 Hz, 1H); MS (ESI): *m/z* calcd. forC_18_H_13_FN_3_[M+H]^+^ 290.1, found: 290.3.

*2-(4-Nitrophenyl)-2,3-dihydro-1H-perimidine* (**7**). Yield = 54%; ^1^H-NMR (DMSO-*d_6_*) δ: 5.50 (s, 1H), 6.52 (d, *J* = 7.2 Hz, 2H), 6.99 (s, 2H), 7.01 (d, *J* = 8.4 Hz, 2H), 7.18 (dd, *J* = 7.6 Hz, 8.0 Hz, 2H), 7.85 (d, *J* = 8.8 Hz, 2H), 8.28 (d, *J* = 8.8 Hz, 2H); MS (ESI): *m/z* calcd. for C_17_H_14_N_3_O_2_ [M+H]^+^ 292.1, found: 292.5.

#### 3.1.2. Procedure for the Preparation of Compound **8**

The mixture of compound **7** (100 mg, 0.34 mmol), iron (38.5 mg, 0.69 mmol) and NH_4_Cl (55.2 mg, 1.03 mmol) in the solution of ethanol (2 mL) and water (1 mL) was heated at 90 °C for 3 h. After filtration, the filter cake was washed with EtOAc, concentrated the filtrate, and dried to afford compound **8** (80 mg, 89%), mp 166.4–171.9 °C. ^1^H-NMR (DMSO-*d*_6_) *δ*: 5.17 (s, 1H), 6.47 (d, *J* = 8.0 Hz, 2H), 6.53 (s, 2H), 6.62 (d, *J* = 8.0 Hz, 2H), 6.96 (d, *J* = 8.0 Hz, 2H), 7.13 (dd, *J* = 7.6 Hz, 8.0 Hz, 2H), 7.26 (d, *J* = 8.0 Hz, 2H); MS (ESI): *m/z* calcd. for C_17_H_16_N_3_[M+H]^+^ 262.1, found: 262.1.

#### 3.1.3. General Procedure for the Preparation of Derivatives **9**–**19**

To a stirred solution of naphthalene-1,8-diamine(500 mg, 3.16 mmol)in methanol (10 mL) was added a solution of 4-formylbenzoic acid methyl ester (621.6 mg, 3.79 mmol) in methanol (5 mL), followed by Zn(OAc)_2_ (58.2 mg, 0.26 mmol). The mixture was stirred at room temperature for 16 h. After filtration, the filter cake was washed with methanol, dried to give compound **9** (300 mg, 31%), mp 165.0–168.2 °C. ^1^H-NMR (CDCl_3_) *δ*: 3.95 (s, 3H), 4.52 (s, 2H), 5.54 (s, 1H), 6.56 (dd, *J* = 1.6 Hz, 6.8 Hz, 2H), 7.24–7.30 (m, 4H), 7.72 (d, *J* = 8.0 Hz, 2H), 8.11 (d, *J* = 8.0 Hz, 2H); MS (ESI): *m/z* calcd. for C_19_H_17_N_2_O_2_ [M+H]^+^ 305.1, found: 305.2.

LiOH·H_2_O (43.5 mg, 0.99 mmol) was added to a solution of compound **9** (100 mg, 0.33 mmol) in THF (1 mL)/H_2_O (1 mL). The mixture was stirred at room temperature for 3 h. After removal of THF, the water layer was washed with EtOAc, and acidified with HCl (1 M) to pH = 2, filtered and dried to get compound **1** (50 mg, 53%), mp > 265 °C; ^1^H-NMR (DMSO-*d*_6_) *δ*: 5.45 (s, 1H), 6.51 (d, *J* = 7.2 Hz, 2H), 6.87 (s, 2H), 7.00 (d, *J* = 8.0 Hz, 2H), 7.17 (dd, *J* = 7.6 Hz, 8.0 Hz, 2H), 7.72 (d, *J* = 8.4 Hz, 2H), 7.99 (d, *J* = 8.0 Hz, 2H), 12.93 (brs, 1 H); MS (ESI): *m/z* calcd. forC_18_H_15_N_2_O_2_[M+H]^+^ 291.1, found: 291.0.

To a stirred solution of compound **1** (1.0 g, 3.4 mmol) in DMF (10 mL) was added methyl glycinate (0.3 g, 3.4 mmol), followed by EDCI (1.0 g, 5.2 mmol) and DMAP (0.04 g, 0.34 mmol). The mixture was stirred at 40 °C overnight. The reaction was diluted with EtOAc (100 mL), washed with water (200 mL × 3). The combined organic phases were then processed in the usual way and chromatographed (2:1 petroleum ether/EtOAc) to yield compound **11** (0.5 g, 40%), mp 193.3–198.7 °C; ^1^H-NMR (DMSO-*d*_6_) *δ*: 3.68 (s, 3H), 4.04 (d, *J* = 6.0 Hz, 2H), 5.44 (s, 1H), 6.51 (d, *J* = 7.6 Hz, 2H), 6.85 (s, 2H), 7.00 (d, *J* = 8.0 Hz, 2H), 7.17 (dd, *J* = 7.6 Hz, 8.0 Hz, 2H), 7.70 (d, *J* = 8.0 Hz, 2H), 7.92 (d, *J* = 8.4 Hz, 2H), 8.99 (t, *J* = 6.4 Hz, 1H); MS (ESI): *m/z* calcd. for C_21_H_20_N_3_O_3_ [M+H]^+^ 362.2, found: 362.1.

LiOH·H_2_O (69.8 mg, 1.66 mmol) was added to a solution of compound **11** (200 mg, 0.55 mmol) in THF (2 mL)/H_2_O (2 mL). The reaction was stirred at room temperature for 3 h. After removal of THF, the water layer was washed with EtOAc, acidified with HCl (1 M) to pH = 2, filtered and dried to afford compound **16** (80 mg, 42%), mp 85.4–90.3 °C;^1^H-NMR (DMSO-*d*_6_) *δ*: 3.93 (d, *J* = 6.0 Hz, 2H), 5.42 (s, 1H), 6.50 (d, *J* = 7.2 Hz, 2H), 6.83 (s, 2H), 6.98 (d, *J* = 8.0 Hz, 2H), 7.17 (t, *J* = 7.6 Hz, 2H), 7.68 (d, *J* = 8.4 Hz, 2H), 7.90 (d, *J* = 8.4 Hz, 2 H), 8.86 (t, *J* = 6.0 Hz, 1H), 12.60 (brs, 1H); ^13^C-NMR (DMSO-*d*_6_) *δ*: 41.2, 65.7, 104.4, 112.0, 115.3, 126.8, 127.2, 127.7, 133.9, 134.3, 142.7, 145.1, 166.7, 171.2; MS (ESI): *m/z* calcd. forC_20_H_18_N_3_O_3_[M+H]^+^ 348.1, found: 348.5.

*Methyl 1-(4-(2,3-dihydro-1H-perimidin-2-yl)benzoyl)pyrrolidine-2-carboxylate* (**10**). Yield = 41%, mp 171.3–176.0 °C; ^1^H-NMR (CDCl_3_) δ:1.93 (m, 1H), 2.04 (m, 2H), 2.34 (m, 1H), 3.55 (m, 1H), 3.64 (m, 1H), 3.80 (s, 3H), 4.53 (s, 2H), 4.69 (dd, *J* = 4.8 Hz, 8.4 Hz, 1H), 5.51 (s, 1H), 6.55 (d, *J* = 6.8 Hz, 2H), 7.27 (m, 4H), 7.65 (d, *J* = 8.0 Hz, 2H), 7.69 (d, *J* = 8.0 Hz, 2H); MS (ESI): *m/z* calcd. for C_24_H_24_N_3_O_3_ [M+H]^+^ 402.2, found: 401.7.

*Ethyl 4-(4-(2,3-dihydro-1H-perimidin-2-yl)benzamido)butanoate* (**12**). Yield = 35%, mp 128.6–132.5 °C; ^1^H-NMR (DMSO-*d_6_*) δ: 1.16 (t, *J* = 7.2 Hz, 3H), 1.78 (m, 2H), 2.35 (t, *J* = 7.2 Hz, 2H), 3.27 (m, 2H), 4.04 (q, *J* = 7.2 Hz, 2H), 5.42 (s, 1H), 6.49 (d, *J* = 8.0 Hz, 2H), 6.83 (s, 2H), 6.98 (d, *J* = 8.0 Hz, 2H), 7.15 (dd, *J* = 7.6 Hz, 8.0 Hz, 2H), 7.65 (d, J = 8.0 Hz, 2H), 7.87 (d, *J* = 8.0 Hz, 2H), 8.50 (t, *J* = 5.2 Hz, 1H); MS (ESI): *m/z* calcd. forC_24_H_26_N_3_O_3_ [M+H]^+^ 404.2, found: 404.0.

*Ethyl 5-(4-(2,3-dihydro-1H-perimidin-2-yl)benzamido)pentanoate* (**13**). Yield = 52%, mp 141.8–143.1 °C; ^1^H-NMR (DMSO-*d_6_*) δ: 1.17 (t, *J* = 7.2 Hz, 3H), 1.54 (m, 4H), 2.32 (t, *J* = 6.8 Hz, 2H), 3.26 (m, 2H), 4.04 (q, *J* = 7.2 Hz, 2H), 5.41 (s, 1H), 6.49 (d, J = 7.6 Hz, 2H), 6.83 (s, 2H), 6.98 (d, *J* = 8.0 Hz, 2H), 7.15 (dd, *J* = 7.6 Hz, 8.0 Hz, 2H), 7.65 (d, *J* = 8.0 Hz, 2H), 7.86 (d, *J* = 8.0 Hz, 2H), 8.48 (t, *J* = 5.2 Hz, 1H); MS (ESI): *m/z* calcd. for C_25_H_28_N_3_O_3_ [M+H]^+^ 418.2, found: 418.1.

*Ethyl 6-(4-(2,3-dihydro-1H-perimidin-2-yl)benzamido)hexanoate* (**14**), Yield = 48%, mp 145.8-147.4 °C; ^1^H-NMR (DMSO-*d_6_*) δ: 1.16 (t, *J* = 7.2 Hz, 3H), 1.23-1.34 (m, 2H), 1.48-1.57 (m, 4H), 2.28 (t, *J* = 7.2 Hz, 2H), 3.23(m, 2H), 4.04 (q, *J* = 7.2 Hz, 2H), 5.42 (s, 1H), 6.49 (d, *J* = 7.6 Hz, 2H), 6.83(s, 2H), 6.98 (d, *J* = 8.0 Hz, 2H), 7.15 (dd, *J* = 7.6 Hz, 8.0 Hz, 2H), 7.65 (d, *J* = 8.0 Hz, 2H), 7.86 (d, *J* = 8.0 Hz, 2H), 8.46 (t, *J* = 5.2 Hz, 1H); MS (ESI): *m/z* calcd. for C_26_H_30_N_3_O_3_ [M+H]^+^ 432.2, found: 432.2.

*1-(4-(2,3-Dihydro-1H-perimidin-2-yl)benzoyl)pyrrolidine-2-carboxylic acid* (**15**). Yield = 42%, mp 233.1–234.3 °C; ^1^H-NMR (DMSO-*d_6_*) δ: 1.91 (m, 4H), 2.29 (m, 2H), 4.42 (m, 1H), 5.42 (s, 1H), 6.51 (d, *J* = 8.0 Hz, 2H), 6.84 (s, 2H), 7.00 (d, *J* = 8.0 Hz, 2H), 7.16 (dd, *J* = 7.6 Hz, 8.0 Hz, 2H), 7.58 (d, *J* = 7.6 Hz, 2H), 7.67 (d, *J* = 8.0 Hz, 2H); MS (ESI): *m/z* calcd. for C_23_H_22_N_3_O_3_ [M+H]^+^ 388.2, found: 388.2.

*4-(4-(2,3-Dihydro-1H-perimidin-2-yl)benzamido)butanoic acid* (**17**). Yield = 55%, mp 99.7–103.3 °C; ^1^H-NMR (DMSO-*d_6_*) δ: 1.75 (m, 2H), 2.27 (m, 2H), 3.27 (m, 2H), 5.42 (s, 1H), 6.49 (d, *J* = 7.2 Hz, 2H), 6.83(s, 2H), 6.98 (d, *J* = 8.0 Hz, 2H), 7.15 (dd, *J* = 7.6 Hz, 8.0 Hz, 2H), 7.65 (d, *J* = 8.4 Hz, 2H), 7.87 (d, *J* = 8.0 Hz, 2H), 8.51 (t, *J* = 5.2 Hz, 1H); ^13^C-NMR (DMSO-*d_6_*) δ: 24.5, 31.2, 38.7, 65.7, 104.4, 112.4, 115.3, 126.8, 127.1, 127.6, 134.3, 134.6, 142.7, 144.8, 165.6, 174.2; MS (ESI): *m/z* calcd. for C_22_H_22_N_3_O_3_ [M+H]^+^ 376.2, found: 376.1.

*5-(4-(2,3-Dihydro-1H-perimidin-2-yl)benzamido)pentanoic acid* (**18**). Yield = 47%, mp 102.8–107.4 °C; ^1^H-NMR (DMSO-*d_6_*) δ: 1.55 (m, 4H), 2.22–2.28 (m, 2H), 3.25 (m, 2H), 5.42 (s, 1H), 6.50 (d, *J* = 7.2 Hz, 2H), 6.98 (d, *J* = 8.0 Hz, 2H), 7.15 (dd, *J* = 7.6 Hz, 8.0 Hz, 2H), 7.65 (d, *J* = 8.0 Hz, 2H), 7.87 (d, *J* = 8.0 Hz, 2H), 8.49 (t, *J* = 5.2 Hz, 1H); ^13^C-NMR (DMSO-*d_6_*) δ: 22.0, 28.6, 33.3, 38.8, 65.8, 104.7, 112.6, 115.6, 126.8, 127.0, 127.7, 134.3, 134.7, 142.4, 144.4, 165.7, 174.4; MS (ESI): *m/z* calcd. for C_23_H_24_N_3_O_3_ [M+H]^+^ 390.2, found: 390.1.

*6-(4-(2,3-Dihydro-1H-perimidin-2-yl)benzamido)hexanoic acid* (**19**). Yield = 50%, mp 90.8–91.9 °C; ^1^H-NMR (DMSO-*d_6_*) δ: 1.30 (m, 2H), 1.48–1.56 (m, 4H), 2.21 (t, *J* = 7.2 Hz, 2H), 3.24 (m, 2H), 5.41 (s, 1H), 6.49 (d, *J* = 8.0 Hz, 2H), 6.83 (s, 2H), 6.98 (d, *J* = 8.0 Hz, 2H), 7.15 (dd, *J* = 7.6 Hz, 8.0 Hz, 2H), 7.65 (d, *J* = 8.0 Hz, 2H), 7.86 (d, *J* = 8.4 Hz, 2H), 8.46 (t, *J* = 5.2 Hz, 1H);^13^C-NMR (DMSO-*d_6_*) δ: 24.2, 26.0, 28.8, 33.6, 39.0, 65.7, 104.4, 112.5, 115.3, 126.8, 127.0, 127.6, 134.3, 134.7, 142.7, 144.7, 165.7, 174.4; MS (ESI): *m/z* calcd. forC_24_H_26_N_3_O_3_ [M+H]^+^ 404.2, found: 404.5.

#### 3.1.4. General Procedure for the Preparation of Derivatives **21**–**25**

To a stirred solution of compound **5** (50 mg, 0.19 mmol) in DMF (2 mL) was added ethyl 4-bromobutyrate (44.6 mg, 0.23 mmol) and cesium carbonate (74.9 mg, 0.23 mmol). Then the resulting mixture was stirred at 40 °C overnight. The reaction was diluted with EtOAc (100 mL), washed with water (100 mL × 3). The organic phase was processed in the usual way and chromatographed (1:1 petroleum ether/EtOAc) to yield compound **21** (41 mg, 65%), mp 76.2–78.6 °C; ^1^H-NMR (DMSO-*d*_6_) *δ*: 1.18 (t, *J* = 7.2 Hz, 3H), 1.96 (t, *J* = 6.4 Hz , 2H), 2.45 (m, 2H), 4.00 (t, *J* = 6.4 Hz, 2H), 4.07 (q, *J* = 7.2 Hz, 2H), 5.29 (s, 1H), 6.47 (d, *J* = 7.2 Hz, 2H), 6.66 (s, 2H), 6.96 (d, *J* = 8.0 Hz, 4H), 7.13 (dd, *J* = 7.6 Hz, 8.0 Hz, 2H), 7.50 (d, *J* = 8.4 Hz, 2H); MS (ESI): *m/z* calcd. for C_23_H_25_N_2_O_3_ [M+H]^+^ 377.2, found: 377.6.

The mixture of LiOH·H_2_O (67.0 mg, 1.60 mmol) and compound **21** (200 mg, 0.53 mmol) in THF (2 mL) and H_2_O (2 mL) was stirred at room temperature for 3 h. After removal of THF, the water layer was washed with EtOAc, acidified with HCl (1 M) to pH 2, filtered and dried to yield compound **24** (100 mg, 54%), mp 126.3–130.9 °C; ^1^H-NMR (DMSO-*d*_6_) *δ*: 1.94 (m, 2H), 2.39 (t, *J* = 7.2 Hz, 2H), 4.02 (t, *J* = 7.2 Hz, 2H), 5.36 (s, 1H), 6.60 (d, *J* = 7.2 Hz, 2H), 7.00 (d, *J* = 8.8 Hz, 2H), 7.09 (d, *J* = 8.0 Hz, 2H), 7.20 (dd, *J* = 7.6 Hz, 8.0 Hz, 2H), 7.55 (d, *J* = 8.4 Hz, 2H); MS (ESI): *m/z* calcd. for C_21_H_21_N_2_O_3_ [M+H]^+^ 349.2, found: 349.3. The following compounds were similarly prepared:

*Ethyl 5-[4-(2,3-dihydro-1H-perimidin-2-yl)phenoxy]pentanoate* (**22**). Yield = 40%, mp 93.8–96.4 °C; ^1^H-NMR (DMSO-*d_6_*) δ: 1.18 (t, *J* = 7.2 Hz, 3H),1.65–1.72 (m, 4H), 2.37 (t, *J* = 7.2 Hz, 2H), 3.98 (t, *J* = 6.0 Hz, 2H), 4.06 (q, *J* = 7.2 Hz, 2H), 5.29 (s, 1H), 6.47 (d, *J* = 7.2 Hz, 2H), 6.67 (s, 2H), 6.96 (d, *J* = 8.0 Hz, 4H), 7.13 (dd, *J* = 7.6 Hz, 8.0 Hz, 2H), 7.51 (d, *J* = 8.4 Hz, 2H); MS (ESI): *m/z* calcd. for C_24_H_27_N_2_O_3_ [M+H]^+^ 391.2, found: 391.3.

*2-[4-(2,3-Dihydro-1H-perimidin-2-yl)phenoxy]acetic acid* (**23**). Yield = 35%, mp > 265 °C; ^1^H-NMR (DMSO-*d_6_*) δ: 4.33 (m, 2H), 5.27 (s, 1H), 6.47 (d, *J* = 7.6 Hz, 2H), 6.66 (s, 2H), 6.89 (d, *J* = 8.4 Hz, 2H), 6.97 (d, *J* = 8.0 Hz, 2H), 7.14 (dd, *J* = 7.6 Hz, 8.0 Hz, 2H), 7.47 (d, *J* = 8.8 Hz, 2H); ^13^C-NMR (DMSO-*d_6_*) δ: 66.1, 67.0, 104.2, 114.3, 115.1, 126.8, 128.2, 128.8, 135.0, 143.3, 152.2, 158.8, 160.9; MS (ESI): *m/z* calcd. for C_19_H_17_N_2_O_3_ [M+H]^+^ 321.1, found: 321.1.

*5-[4-(2,3-Dihydro-1H-perimidin-2-yl)phenoxy]pentanoic acid* (**25**). Yield = 50%, mp 165.9–167.8 °C; ^1^H-NMR (DMSO-*d_6_*) δ: 1.63–1.75 (m, 4H), 2.29 (t, *J* = 7.2 Hz, 2H), 3.99 (t, *J* = 6.4 Hz, 2H), 5.32 (s, 1H), 6.52 (d, *J* = 7.2 Hz, 2H), 7.01 (m, 4H), 7.16 (t, *J* = 7.6 Hz, 2H), 7.51 (d, *J* = 8.8 Hz, 2H); MS (ESI): *m/z* calcd. for C_22_H_23_N_2_O_3_ [M+H]^+^ 363.2, found: 363.3.

#### 3.1.5. General Procedure for the Preparation of Derivatives **26**–**33**

A mixture of compound **8** (50 mg, 0.19 mmol), succinic acid monoethyl ester (27.7 mg, 0.19 mmol), EDCI (55.2 mg, 28.7 mmol) and DMAP (2.3 mg, 0.019 mmol) in DMF (2 mL) was stirred at 40 °C overnight. The reaction mixture was diluted with EtOAc (100 mL), washed with water (50 mL × 3), the organic layer was then processed in the usual way and chromatographed (1:1 petroleum ether/EtOAc) to yield compound **28** (30 mg, 41%), mp 165.8–170.9 °C; ^1^H-NMR (DMSO-*d*_6_) *δ*: 1.18 (t, *J* = 7.2 Hz, 3H), 2.57–2.63 (m, 4H), 4.06 (q, *J* = 7.2 Hz, 2H), 5.29 (s, 1H), 6.48 (d, *J* = 8.0 Hz, 2H), 6.67 (s, 2H), 6.97 (d, *J* = 8.0 Hz, 2H), 7.14 (dd, *J* = 7.6 Hz, 8.0 Hz, 2H), 7.51 (d, *J* = 8.4 Hz, 2H), 7.61 (d, *J* = 8.4 Hz, 2H); MS (ESI): *m/z* calcd. for C_23_H_24_N_3_O_3_ [M+H]^+^ 390.2, found: 390.0.

A mixture of compound **28** (30 mg, 0.077 mmol) and LiOH·H_2_O (9.7 mg, 0.23 mmol) in THF (2 mL) and H_2_O (2 mL) was stirred at room temperature for 3 h. After removal of THF, the water layer was washed with EtOAc, acidified with HCl (1 M) to pH 2, filtered and dried to get compound **32** (15 mg, 54%), mp > 265 °C; ^1^H-NMR (DMSO-*d*_6_) *δ*: 2.50–2.56 (m, 4H), 5.29 (s, 1H), 6.48 (d, *J* = 8.0 Hz, 2H), 6.67 (s, 2H), 6.97 (d, *J* = 8.0 Hz, 2H), 7.14 (dd, *J* = 7.6 Hz, 8.0 Hz, 2H), 7.50 (d, *J* = 8.4 Hz, 2H), 7.61 (d, *J* = 8.4 Hz, 2H), 10.14 (s, 1H), 12.10 (brs, 1H); MS (ESI): *m/z* calcd. for C_21_H_20_N_3_O_3_ [M+H]^+^ 362.2, found: 362.2. The following compounds were similarly prepared:

*Ethyl 2-{[4-(2,3-dihydro-1H-perimidin-2-yl)phenyl]amino}-2-oxoacetate* (**26**). Yield = 41%, mp 132.3–135.5 °C; ^1^H-NMR (DMSO-*d_6_*) δ: 1.32 (t, *J* = 7.2 Hz, 3H), 4.31 (q, *J* = 7.2 Hz, 2H), 5.32 (s, 1H), 6.48 (d, *J* = 7.6 Hz, 2H), 6.73 (s, 2H), 6.97 (d, *J* = 8.0 Hz, 2H), 7.14 (dd, *J* =7.6 Hz, 8.0 Hz, 2H), 7.57 (d, *J* = 8.4 Hz, 2H), 7.77 (d, *J* = 8.8 Hz, 2H), 10.86 (s, 1H); ^13^C-NMR (DMSO-*d_6_*) δ:13.8, 62.4, 65.9, 104.3, 112.4, 115.2, 120.3, 126.8, 128.3, 134.4, 137.6, 138.1, 143.0, 155.6, 160.7; MS (ESI): *m/z* calcd. for C_21_H_20_N_3_O_3_ [M+H]^+^ 362.1, found: 362.0.

*Ethyl 3-{[4-(2,3-dihydro-1H-perimidin-2-yl)phenyl]amino}-3-oxopropanoate* (**27**). Yield = 51%, mp 158.2–160.7 °C; ^1^H-NMR (DMSO-*d_6_*) δ: 1.21 (t, *J* = 7.2 Hz, 3H), 3.47 (s, 2H), 4.12 (q, *J* = 7.2 Hz, 2H), 5.30 (s, 1H), 6.48 (d, *J* = 8.0 Hz, 2H), 6.69 (s, 2H), 6.97 (d, *J* = 8.0 Hz, 2H), 7.14 (dd, *J* = 7.6 Hz, 8.0 Hz, 2H), 7.53 (d, *J* = 8.0 Hz, 2H), 7.61 (d, *J* = 8.4 Hz, 2H); MS (ESI): *m/z* calcd. for C_22_H_22_N_3_O_3_ [M+H]^+^ 376.2, found: 375.6.

*Ethyl 5-{[4-(2,3-dihydro-1H-perimidin-2-yl]phenyl)amino}-5-oxopentanoate* (**29**). Yield = 35%, mp 139.9–142.5 °C; ^1^H-NMR (DMSO-*d_6_*) δ: 1.19 (t, *J* = 7.2 Hz, 3H), 1.84 (m, 2H), 2.36 (m, 4H), 4.06 (q, *J* = 7.2 Hz, 2H), 5.29 (s, 1H), 6.48 (d, *J* = 7.2 Hz, 2H), 6.67 (s, 2H) , 6.97 (d, *J* = 8.0 Hz, 2H), 7.14 (t, *J* = 7.6 Hz, 2H), 7.51 (d, *J* = 8.4 Hz, 2H), 7.62 (d, *J* = 8.4 Hz, 2H), 9.97 (s, 1H); MS (ESI): *m/z* calcd. for C_24_H_26_N_3_O_3_ [M+H]^+^ 404.3, found: 404.1.

*2-{[4-(2,3-Dihydro-1H-perimidin-2-yl)phenyl]amino}-2-oxoacetic acid* (**30**). Yield = 35%, mp > 265 °C; ^1^HNMR (400MHz, DMSO-*d_6_*) δ: 5.32 (s, 1H), 6.48 (d, *J* = 7.2 Hz, 2H), 6.73(s, 2H), 6.97 (d, *J* = 8.4 Hz, 2H), 7.14 (dd, *J* = 7.6 Hz, 8.0 Hz, 2H), 7.56 (d, *J* = 8.4 Hz, 2H), 7.78 (d, *J* = 8.4 Hz, 2H), 10.79 (s, 1H).

*3-{[4-(2,3-Dihydro-1H-perimidin-2-yl)phenyl]amino}-3-oxopropanoic acid* (**31**). Yield = 52%, mp 221.3–223.4 °C; ^1^H-NMR (DMSO-*d_6_*) δ: 3.37 (s, 2H), 5.32 (s, 1H), 6.51 (d, *J* = 7.2 Hz, 2H), 7.00 (d, *J* = 8.0 Hz, 2H), 7.15 (dd, *J* = 7.6 Hz, 8.0 Hz, 2H), 7.54 (d, *J* = 8.4 Hz, 2H), 7.62 (d, *J* = 8.4 Hz, 2H), 10.24 (s, 1H); ^13^C-NMR (DMSO-*d_6_*) δ: 43.9, 66.3, 105.1, 112.7, 115.9, 118.4, 118.7, 126.8, 128.5, 134.3, 139.3, 142.3, 164.6, 169.2; MS (ESI): *m/z* calcd. for C_20_H_18_N_3_O_3_ [M+H]^+^ 348.1, found: 348.0.

*5-{[4-(2,3-Dihydro-1H-perimidin-2-yl)phenyl]amino}-5-oxopentanoic acid* (**33**). Yield = 56%, mp > 265 °C; ^1^H-NMR (DMSO-*d_6_*) δ: 1.80 (m, 2H), 2.26–2.45 (m, 4H), 5.29 (s, 1H), 6.48 (d, *J* = 7.2 Hz, 2H), 6.97 (d, *J* = 8.0 Hz, 2H), 7.14 (dd, *J* = 7.6 Hz, 8.0 Hz, 2H),7.50 (d, *J* = 8.8 Hz, 2H), 7.62 (d, *J* = 8.8 Hz, 2H), 10.00 (s, 1H); MS (ESI): *m/z* calcd. for C_22_H_22_N_3_O_3_ [M+H]^+^ 376.2, found: 376.1.

#### 3.1.6. Procedure for the Preparation of Compound **34** and **35**

A mixture of compound **8** (100 mg, 0.38 mmol), 3-*tert*-butoxycarbonylaminopropionic acid (72 mg, 0.38 mmol), EDCI (110 mg, 57 mmol) and DMAP (4.6 mg, 0.038 mmol) in DMF (4 mL) was stirred at 40 °C overnight. The reaction mixture was diluted with EtOAc (100 mL), washed with water (50 mL × 3), the organic layer was then processed in the usual way and chromatographed (2:1 petroleum ether/EtOAc) to yield compound **34** (72 mg, 43%), mp 111.1–113.7 °C; ^1^H-NMR (DMSO-*d*_6_) *δ*: 1.38 (s, 9H), 2.48 (m, 2H), 3.21 (m, 2H), 5.29 (s, 1H), 6.48 (d, *J* = 7.2 Hz, 2H), 6.68 (s, 2H), 6.90 (m, 1H), 6.97 (d, *J* = 8.0 Hz, 2H), 7.14 (dd, *J* = 7.6 Hz, 8.0 Hz, 2H), 7.51 (d, *J* = 8.4 Hz, 2H), 7.62 (d, *J* = 8.4 Hz, 2H), 10.01 (s, 1H); MS (ESI): *m/z* calcd. for C_25_H_29_N_4_O_3_ [M+H]^+^ 433.2, found: 433.1.

Trifluoroacetic acid (1 mL) was added slowly to the solution of compound **34** (50 mg, 0.12 mmol) in DCM (5 mL) at 0 °C. After stirred at room temperature for 5 h, the solution was concentrated to yield compound **35** (26 mg, 67%), mp 239.7–241.2 °C; ^1^H-NMR (DMSO-*d*_6_) *δ*: 2.72 (m, 2H), 3.12 (m, 2H), 5.31 (s, 1H), 6.48 (d, *J* = 7.2 Hz, 2H), 6.69 (s, 2H), 6.97 (d, *J* = 7.6 Hz, 2H), 7.14 (dd, *J* = 7.6 Hz, 8.0 Hz, 2H), 7.53 (d, *J* = 8.4 Hz, 2H), 7.63 (d, *J* = 8.8 Hz, 2H), 7.80 (brs, 2H), 10.25 (s, 1H); MS (ESI): *m/z* calcd. for C_20_H_21_N_4_O [M+H]^+^ 333.2, found: 333.0.

#### 3.1.7. Procedure for the Preparation of Compounds **40**–**49**

SOCl_2_ (28.5 mL, 0.24 mol) was added slowly to a stirred solution of compound **36** (10 g, 59.9 mmol) in methanol (100 mL) at 0 °C. After the addition, the solution was stirred at 80 °C for 4 h and then concentrated via rotary evaporator to get compound **37** (11.4 g, 98%). NaBH_4_ (2.4 g, 64 mmol) was added dropwise to the mixture of compound **37** (5 g, 25.6 mmol) and CaCl_2_ (11.4 g, 102.6 mmol) in THF (25 mL)/EtOH (25 mL) at 0 °C. After completion, the reaction was quenched with water. The aqueous phase was extracted with EtOAc. The combined organic phases were then processed in the usual way and chromatographed (1:1 petroleum ether/EtOAc) to yield compound **38** (2.5 g, 58%). Dess-Martin periodinane (3.0 g, 7.2 mmol) was added slowly to the mixture of compound **38** (1.0 g, 6.0 mmol) in DCM (10 mL). The resulting mixture continued to stir at room temperature overnight. The reaction was quenched with water. The aqueous phase was extracted with EtOAc. The combined organic phases were then processed in the usual way and chromatographed (3:1 petroleum ether/EtOAc) to yield compound **39** (0.85 g, 86%). To a stirred solution of compound **39** (0.3 g, 1.82 mmol) in methanol (5 mL) was added a solution of naphthalene-1,8-diamine (0.24 g, 1.52 mmol) in methanol (5 mL). Then Zn(OAc)_2_ (0.028 g, 0.128 mmol) was added and the mixture was stirred at room temperature for 16 h. The reaction mixture was filtered, the filter cake was washed with methanol, dried to get compound **40** (125 mg, 27%), mp 92.3–94.8 °C. ^1^H-NMR (DMSO-*d*_6_) *δ*: 3.88 (s, 3H), 5.50 (s, 1H), 6.54 (d, *J* = 7.6 Hz, 2H), 6.98 (d, *J* = 8.0 Hz, 2H), 7.08 (s, 2H), 7.16 (dd, *J* = 7.6 Hz, 8.0 Hz, 2H), 7.67 (d, *J* = 8.0 Hz, 1H), 8.31 (dd, *J* = 2.0 Hz, 8.0 Hz, 1H), 9.08 (d, *J* = 2.0 Hz, 1H); MS (ESI): *m/z* calcd. for C_18_H_16_N_3_O_2_ [M+H]^+^ 306.1, found: 306.2.

LiOH·H_2_O (21.6 mg, 0.49 mmol) was added to a solution of compound **40** (50 mg, 0.16 mmol) in THF (2 mL) and H_2_O (1 mL), then the mixture was stirred at room temperature for 3 h. After removal of the THF, the water layer was washed with EtOAc, acidified with HCl (1 M) to pH 2, filtered and dried to yield compound **41** (20 mg, 43%), mp 100.7–101.3 °C. ^1^H-NMR (DMSO-*d*_6_) *δ*: 5.50 (s, 1H), 6.55 (d, *J* = 7.2 Hz, 2H), 6.98 (d, *J* = 8.0 Hz, 2H), 7.16 (dd, *J* = 7.6 Hz, 8.0 Hz, 2H), 7.66 (d, *J* = 8.0 Hz, 1H), 8.29 (dd, *J* = 2.0 Hz, 8.0 Hz, 1H), 9.07 (d, *J* = 1.2 Hz, 1H); ^13^C-NMR (DMSO-*d*_6_) *δ*: 66.2, 104.9, 112.3, 115.6, 121.0, 126.2, 127.0, 134.2, 138.1, 141.3, 149.4, 164.8, 165.9; MS (ESI): *m/z* calcd. for C_17_H_14_N_3_O_2_ [M+H]^+^ 292.1, found: 292.5.

To a stirred solution of compound **41** (500 mg, 1.72 mmol) in DMF (10 mL) was added methyl glycinate (230 mg, 2.6 mmol), followed by EDCI (500 mg, 2.5 mmol) and DMAP (21 mg, 0.17 mmol). The mixture was stirred at 40 °C overnight. The reaction was diluted with EtOAc (100 mL), washed with water (200 mL × 3). The combined organic phases were then processed in the usual way and chromatographed (2:1 petroleum ether/EtOAc) to yield compound **42** (249 mg, 40%), mp 183.6–184.7 °C. ^1^H-NMR (DMSO-*d*_6_) *δ*: 3.66 (s, 3H), 4.04 (d, *J* = 6.0 Hz, 2H), 5.49 (s, 1H), 6.55 (d, *J* = 7.2 Hz, 2H), 6.98 (d, *J* = 8.0 Hz, 2H), 7.04 (s, 2H), 7.16 (dd, *J* = 7.6 Hz, 8.0 Hz, 2H), 7.65 (d, *J* = 8.0 Hz, 1H), 8.21 (dd, *J* = 2.0 Hz, 8.0 Hz, 1H), 9.01 (d, *J* = 1.6 Hz, 1H), 9.19 (t, *J* = 5.6 Hz, 1H); MS (ESI): *m/z* calcd. for C_20_H_19_N_4_O_3_ [M+H]^+^ 363.1, found: 363.3.

LiOH·H_2_O (52 mg, 1.2mmol) was added to a solution of compound **42** (150 mg, 0.41 mmol) in THF (2 mL)/H_2_O (2 mL).The reaction was stirred at room temperature for 3 h. After removal of THF, the water layer was washed with EtOAc, acidified with HCl (1 M) to pH = 2, filtered and dried to get compound **46** (60 mg, 42%), mp 103.5–107.3 °C. ^1^H-NMR (DMSO-*d*_6_) *δ*: 3.95 (d, *J* = 6.0 Hz, 2H), 5.48 (s, 1H), 6.55 (d, *J* = 7.6 Hz, 2H), 6.98 (d, *J* = 8.4 Hz, 2H), 7.04 (s, 2H), 7.16 (dd, *J* = 7.6 Hz, 8.4 Hz, 2H), 7.65 (d, *J* = 8.4 Hz, 1H), 8.22 (dd, *J* = 2.0 Hz, 8.4 Hz, 1H), 9.01 (d, *J* = 1.6 Hz, 1H), 9.08 (t, *J* = 6.0 Hz, 1H), 12.68 (brs, 1H); ^13^C-NMR (DMSO-*d*_6_) *δ*: 41.2, 66.4, 104.6, 112.3, 115.4, 120.6, 127.0, 128.8, 134.2, 135.8, 141.7, 147.7, 163.7, 164.9, 171.0; MS (ESI): *m/z* calcd. for C_19_H_17_N_4_O_3_[M+H]^+^ 349.1, found: 349.3. The following compounds were similarly prepared:

*Methyl 4-[6-(2,3-dihydro-1H-perimidin-2-yl)nicotinamido]butanoate* (**43**). Yield = 52%, mp 100.1–105.4 °C; ^1^H-NMR (DMSO-*d_6_*) δ: 1.78 (m, 2H), 2.37 (t, *J* = 7.2 Hz, 2H), 3.28 (m, 2H), 3.57 (s, 3H), 5.47 (s, 1H), 6.54 (d, *J* = 7.2 Hz, 2H), 6.97 (d, *J* = 8.0 Hz, 2H), 7.03 (s, 2H), 7.15 (dd, *J* = 7.6 Hz, 8.0 Hz, 2H), 7.62 (d, *J* = 8.0 Hz, 1H), 8.17 (dd, *J* = 2.0 Hz, 8.0 Hz, 1H), 8.67 (t, *J* = 5.2 Hz, 1H), 8.97 (d, *J* = 1.6 Hz, 1H); MS (ESI): *m/z* calcd. for C_22_H_23_N_4_O_3_ [M+H]^+^ 391.2, found: 391.4.

*Ethyl 5-[6-(2,3-dihydro-1H-perimidin-2-yl)nicotinamido]pentanoate* (**44**). Yield = 60%, mp 109.4–113.5 °C; ^1^H-NMR (DMSO-*d_6_*) δ: 1.16 (t, *J* = 6.8 Hz, 3H), 1.54 (m, 4H), 2.32 (m, 2H), 3.26 (m, 2H), 4.03 (q, *J* = 6.8 Hz, 2H), 5.47 (s, 1H), 6.54 (d, *J* = 7.2 Hz, 2H), 6.97 (d, *J* = 8.0 Hz, 2H), 7.03 (s, 2H), 7.15 (dd, *J* = 7.6 Hz, 8.0 Hz, 2H), 7.62 (d, *J* = 8.4 Hz, 1H), 8.16 (dd, *J*= 2.0 Hz, 8.0 Hz, 1H), 8.65 (t, *J* = 5.6 Hz, 1H), 8.96 (d, *J* = 1.6 Hz, 1H); MS (ESI): *m/z* calcd. for C_24_H_27_N_4_O_3_ [M+H]^+^ 419.2, found: 419.1.

*Ethyl 6-[6-(2,3-dihydro-1H-perimidin-2-yl)nicotinamido]hexanoate* (**45**). Yield = 44%, mp 119.3–123.8 °C; ^1^H-NMR (DMSO-*d_6_*) δ: 1.15 (t, *J* = 7.2 Hz, 3H), 1.23–1.34 (m, 2H), 1.49–1.56 (m, 4H), 2.26–2.30 (m, 2H), 3.22–3.27 (m, 2H), 4.02 (q, *J* = 7.2 Hz, 2H), 5.47 (s, 1H), 6.45 (d, *J* = 7.2 Hz, 2H), 6.97 (d, *J* = 8.0 Hz, 2H), 7.03 (s, 2H), 7.15 (dd, *J* = 7.6 Hz, 8.0 Hz, 2H), 7.61 (d, *J* = 8.0 Hz, 1H), 8.16 (d, *J* = 2.0 Hz, 8.0 Hz, 1H), 8.62 (t, *J* = 5.6 Hz, 1H), 8.96 (d, *J* = 1.6 Hz, 1H); MS (ESI): *m/z* calcd. for C_25_H_29_N_4_O_3_ [M+H]^+^ 433.2, found: 433.2.

*4-[6-(2,3-Dihydro-1H-perimidin-2-yl)nicotinamido]butanoic acid* (**47**). Yield = 50%, mp 95.3–99.0 °C; ^1^H-NMR (DMSO-*d_6_*) δ: 1.72 (m, 2H), 2.21 (t, *J* = 6.8 Hz, 2H), 3.26 (m, 2H), 5.47 (s, 1H), 6.54 (d, *J* = 7.2 Hz, 2H), 6.97 (d, *J* = 8.4 Hz, 2H), 7.02 (s, 2H), 7.15 (t, *J* = 7.6 Hz, 2H), 7.61 (d, *J* = 8.4 Hz, 1H), 8.19 (dd, *J* = 1.6 Hz, 8.0 Hz, 1H), 8.99 (s, 1H), 9.33 (brs, 1H); ^13^C-NMR (DMSO-*d_6_*) δ: 24.6, 32.6, 39.2, 66.3, 104.6, 112.3, 115.4, 120.4, 126.9, 129.5, 134.2, 135.7, 141.7, 147.6, 163.3, 164.4, 175.0; MS (ESI): *m/z* calcd. for C_21_H_21_N_4_O_3_ [M+H]^+^ 377.2, found: 377.1.

*5-[6-(2,3-Dihydro-1H-perimidin-2-yl)nicotinamido]pentanoic acid* (**48**). Yield = 46%, mp 117.1–120.3 °C; ^1^H-NMR (DMSO-*d_6_*) δ: 1.54 (m, 4H), 2.24 (m, 2H), 3.25 (m, 2H), 5.47 (s, 1H), 6.54 (d, *J* = 7.2 Hz, 2H), 6.97 (d, *J* = 8.0 Hz, 2H), 7.02 (s, 2H), 7.15 (t, *J* = 7.6 Hz, 2H), 7.62 (d, *J* = 8.0 Hz, 1H), 8.17 (d, *J* = 8.0 Hz, 1H), 8.65 (t, *J* = 5.2 Hz, 1H), 8.97 (s, 1H), 12.03 (brs, 1H); ^13^C-NMR (DMSO-*d_6_*) δ: 22.1, 28.5, 33.4, 39.0, 66.3, 104.7, 112.4, 115.5, 120.6, 127.0, 129.5, 134.3, 135.8, 141.7, 147.6, 163.4, 164.6, 174.5; MS (ESI): *m/z* calcd. for C_22_H_23_N_4_O_3_ [M+H]^+^ 391.2, found: 391.4.

*6-[6-(2,3-Dihydro-1H-perimidin-2-yl)nicotinamido]hexanoic acid* (**49**). Yield = 48%, mp 86.7–88.4 °C; ^1^H-NMR (DMSO-*d_6_*) δ: 1.23–1.34 (m, 2H), 1.48–1.57 (m, 4H), 2.20 (t, *J* = 7.2 Hz, 2H), 3.24 (m, 2H), 5.48 (s, 1H), 6.55 (d, *J* = 7.2 Hz, 2H), 6.97 (d, *J* = 8.0 Hz, 2H), 7.15 (dd, *J* = 7.6 Hz, 8.0 Hz, 2H), 7.62 (d, *J* = 8.0 Hz, 1H), 8.17 (dd, *J* = 2.0 Hz, 8.0 Hz, 1H), 8.64 (t, *J* = 5.6 Hz, 1H), 8.96 (d, *J* = 1.6 Hz, 1H); ^13^C-NMR (DMSO-*d_6_*) δ:24.3, 26.1, 28.7, 33.7, 39.2, 66.3, 104.8, 112.2, 115.6, 120.3, 127.0, 128.6, 134.9, 135.9, 141.6, 147.8, 163.4, 164.5, 174.5; MS (ESI): *m/z* calcd. for C_23_H_25_N_4_O_3_ [M+H]^+^ 405.2, found: 405.3.

### 3.2. PTP1B and Related PTPs Biological Assay

A colorimetric assay to measure inhibition against PTP1B and TCPTP was performed in 96-well plates. Briefly, the tested compounds were solubilized in DMSO and serially diluted into concentrations for the inhibitory test. The assays were carried out in a final volume of 100 μL containing 50 mmol/L MOPS, pH 6.5, 2 mmol/L pNPP, 30 nmol/L GST-PTP1B or GST-TCPTP,and 2% DMSO, and the catalysis of pNPP was continuously monitored on a SpectraMax 340 microplate reader at 405 nm for 3 min at 30 °C. The IC_50_ value was calculated from the nonlinear curve fitting of the percent inhibition [inhibition (%)] *vs.* the inhibitor concentration using the Equation (1):

Inhibition (%) = 100/[1 + (IC_50_/[I]*k*)] (1)

where *k* is the Hill coefficient. To study the inhibition on the other PTPase family members, SHP1, SHP2 and LAR were prepared and assays were performed according to procedures described previously [24,25]. Briefly, the enzymatic activity of the SHP1, SHP2 and LARwere determined at 30 °C by monitoring the dephosphorylation of substrate 3-o-methylfluorescein phosphate (OMFP), product was then detected at a 485 nm excitation wavelength and 530 nm emission wavelength by the EnVision multilabe plate reader (Perkin-Elmer Life Sciences, Boston, MA, USA). The assays were carried out in a final volume of 50 μL containing 50 mmol/L MOPS, pH 6.8, 10 μmol/L OMFP, 20 nmol/L recombinant enzyme, 2 mmol/L dithiothreitol , 1 mmol/L EDTA, and 2% DMSO. The initial rate of the dephosphorylation was presented by the early linear region of the enzymatic reaction kinetic curve, the inhibitory activity of the compound was continuously monitored.

### 3.3. Characterization of the Inhibitor on Enzyme Kinetics [25]

In the fast-binding inhibition experiment, PTP1B were preincubated with compounds (2% DMSO) on the ice for different times, and then add 10 μL mixture of enzyme and compounds to 90 μL assay system. To characterize the inhibitor of PTP1B, the assay was carried out in a 100 μL system containing 50 mmol/L MOPS, pH 6.5, 14 nmol/L PTP1B, *p*NPP in 2-fold dilution from 80 mmol/L, and different concentrations of the inhibitor. In the presence of the competitive inhibitor, the Michaelis-Menten equation is described as Equation (2):

1/*ν* = [*K*_max_[S])] × (1 + [I]/*K*_i_) + 1/*V*_max_)] (2)

where *K*_m_ is the Michaelis constant, *v* is the initial rate, *V*_max_ is the maximum rate, and [S] is the substrate concentration. The *K*_i_ value was obtained by the linear replot of apparent *K*_m_/*V*_max_ (slope) from the primary reciprocal plot *versus* the inhibitor concentration [I] according to the equation *K*_m_/*V*_max_ = 1 + [I]/*K*_i_.

### 3.4. Cellular Activity of Compound **46**

CHO/*h*IR cells were cultured in F12 nutrient medium including 10% (V/V) FBS, 100 units/mL penicillin and 100 μg/mL streptomycin with 5% CO_2_ at 37 °C. Cells were serum free starved for 2 h, and then treated with compounds for 3 h, followed with insulin (10 nM, Eli Lilly) for 10 min before harvested. Cells were rinsed twice with precooled 1× PBS and then lysed with 1× SDS loading buffer. Samples were heated at 100 °C for 15 min before electrophoresed with 8% SDS-polyarylamide gel under 80 to 120 volt voltage, and then transferred to nitrocellulose (NC) membranes. NC membranes were blocked for 2 h with 5% BSA (W/V) dissolved in TBST. The membranes incubated with primary antibodies overnight at 4 °C and secondary antibodies for 1 h at room temperature. The primary antibody p-Tyr (PY20) used was from Santa Cruz (Dallas, CA, USA) and β-actin from Sigma (St. Louis, MO, USA), secondary antibody was from Jackson Immuno Research (Philadelphia, PA, USA).

## 4. Conclusions

In conclusion, a series of 1*H*-2,3-dihydroperimidine derivatives were synthesized and identified as PTP1B inhibitors with IC_50_ in the micromolar range. After performing systematic SAR studies, we identified two compounds with IC_50_ values less than 1 μM. Among these, the representative compounds had no visible activities against receptor-like transmembrane LAR. Compound **46** possessed about 9-fold selectivity for PTP1B over SHP-1 and SHP-2, respectively. More importantly, compound **46** exhibited 3.48-fold selectivity for PTP1B over TCPTP, and cellular activity for protection of phosphorylation of IR. These results provide a possible opportunity for the development of novel PTP1B inhibitors with promising cell permeability, bioavailability, and improved pharmacological properties. 
